# MRI feature-based radiomics models to predict treatment outcome after stereotactic body radiotherapy for spinal metastases

**DOI:** 10.1186/s13244-023-01523-5

**Published:** 2023-10-10

**Authors:** Yongye Chen, Siyuan Qin, Weili Zhao, Qizheng Wang, Ke Liu, Peijin Xin, Huishu Yuan, Hongqing Zhuang, Ning Lang

**Affiliations:** 1 Department of Radiology, Peking University Third Hospital, 49 North Garden Road, Haidian District, Beijing, 100191 People’s Republic of China; 2 Department of radiotherapy, Peking University Third Hospital, 49 North Garden Road, Haidian District, Beijing, 100191 People’s Republic of China

**Keywords:** Spine, Neoplasm metastasis, MRI, Radiosurgery, Treatment outcome

## Abstract

**Objective:**

This study aimed to extract radiomics features from MRI using machine learning (ML) algorithms and integrate them with clinical features to build response prediction models for patients with spinal metastases undergoing stereotactic body radiotherapy (SBRT).

**Methods:**

Patients with spinal metastases who were treated using SBRT at our hospital between July 2018 and April 2023 were recruited. We assessed their response to treatment using the revised Response Evaluation Criteria in Solid Tumors (version 1.1). The lesions were categorized into progressive disease (PD) and non-PD groups. Radiomics features were extracted from T1-weighted image (T1WI), T2-weighted image (T2WI), and fat-suppression T2WI sequences. Feature selection involved intraclass correlation coefficients, minimal-redundancy-maximal-relevance, and least absolute shrinkage and selection operator methods. Thirteen ML algorithms were employed to construct the radiomics prediction models. Clinical, conventional imaging, and radiomics features were integrated to develop combined models. Model performance was evaluated using receiver operating characteristic (ROC) curve analysis, and the clinical value was assessed using decision curve analysis.

**Results:**

We included 194 patients with 142 (73.2%) lesions in the non-PD group and 52 (26.8%) in the PD group. Each region of interest generated 2264 features. The clinical model exhibited a moderate predictive value (area under the ROC curve, AUC = 0.733), while the radiomics models demonstrated better performance (AUC = 0.745–0.825). The combined model achieved the best performance (AUC = 0.828).

**Conclusion:**

The MRI-based radiomics models exhibited valuable predictive capability for treatment outcomes in patients with spinal metastases undergoing SBRT.

**Critical relevance statement:**

Radiomics prediction models have the potential to contribute to clinical decision-making and improve the prognosis of patients with spinal metastases undergoing SBRT.

**Key points:**

• Stereotactic body radiotherapy effectively delivers high doses of radiation to treat spinal metastases.

• Accurate prediction of treatment outcomes has crucial clinical significance.

• MRI-based radiomics models demonstrated good performance to predict treatment outcomes.

**Graphical Abstract:**

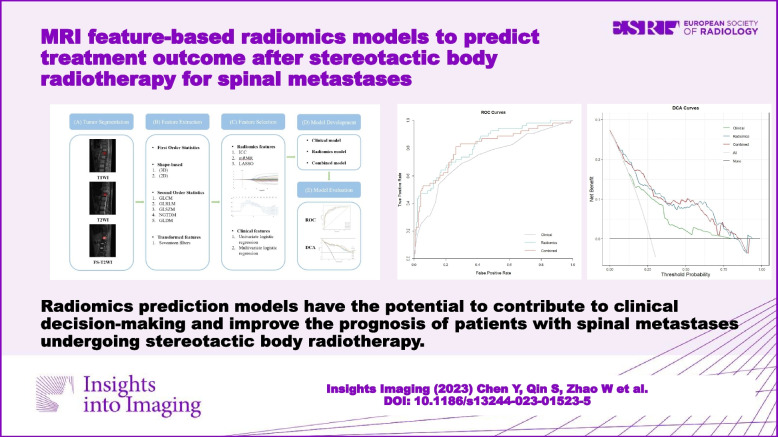

## Introduction

Bone metastases commonly affect the spine [1]. The number of patients presenting with spinal metastases is rising because of increasing cancer incidence and life expectancy [2, 3]. Spinal metastases can lead to pain, vertebral compression fractures, and compression of the spinal cord or nerve roots, which significantly affect patients’ quality of life [4].

The advanced radiotherapy technology, stereotactic body radiotherapy (SBRT), delivers highly conformal and ablative doses to extracranial target lesions [5]. Compared with palliative radiotherapy, SBRT more accurately irradiates spine metastatic lesions using ablative doses, while reducing radiation-induced injury risk to the minimum [6]. The intense antitumor effects of ablative irradiation with SBRT result in a high tumor control rate [7]. Studies suggest that imaging-based local control rates range from 57 to 100% [7–9].

Prediction of outcomes in patients with spinal metastases treated with SBRT could assist in treatment decision-making and in managing prognostic expectations. For those patients who do not achieve local control, the treatment plan may be altered to include systemic therapy and surgery. Previous studies have found that some clinical features such as the primary tumor site, polymetastatic disease, performance status, and pain level are associated with prognosis after SBRT in patients with spinal metastases [10–13]. However, the relationship between imaging features and patient prognosis is unknown.

MRI is a commonly used imaging method before patients receive SBRT and might have the potential for prognostic prediction. MRI has a high soft-tissue resolution, allowing for better visualization of spinal metastases and optimization of the accuracy of target volume delineation for SBRT compared with CT [14]. With recent advances in computer-aided diagnosis, quantitative image methods together with machine learning (ML) algorithms, which can extract large-scale data from medical images, might provide valuable prognostic information [15].

Therefore, in this study, we aimed to apply ML methods to extract high-throughput radiomics features from MRI, selecting optimal radiomics features integrated with clinical features to build a response prediction model for SBRT-treated patients with spinal metastases.

## Materials and methods

### Patient selection

The ethics committee of our hospital approved this prospective study. We recruited consecutive patients with spinal metastases who received SBRT at our institution from July 2018 to April 2023. All patients provided informed consent.

The inclusion criteria comprised: (1) a diagnosis of spinal metastases based on pathological biopsy or imaging, and (2) undergoing MRI within 1 week before receiving SBRT. The exclusion criteria comprised: (1) the patient’s target lesion area had received previous radiotherapy or surgery, (2) the patient had received chemotherapy within 1 month, (3) the patient was unable or refused to cooperate with CT and MRI examinations, (4) the patient was lost to follow-up, or (5) the image quality was poor, making analysis impossible.

### MRI data acquisition

A 3.0-T GE Discovery MR 750 (GE Healthcare) MRI scanner was used, which had a phased-array body coil with eight channels. The following MRI sequences were performed: sagittal T1-weighted image (T1WI) (repetition time (TR): 400–750 ms; echo delay time (TE): 8–25 ms), sagittal T2WI (TR: 2500–4000 ms; TE: 120–140 ms), and sagittal fat-suppression T2WI (FS-T2WI) (TR: 2500–4000 ms; TE: 100–120 ms), transverse T2WI (TR: 2500–4000 ms; TE: 100–120 ms).

### SBRT procedures and treatment outcome assessment

The treatment platform comprised the CyberKnife stereotactic radiotherapy system (Accuray Inc). Before treatment planning CT, patients were immobilized using either the BodyFix system for thoracic, lumbar, and sacral spine lesions, or a customized thermoplastic mask for cervical spine lesions. According to the Consensus Guidelines of the International Spine Radiosurgery Consortium [16], target delineation was conducted on co-registered MRI and CT datasets. A physicist formulated the radiotherapy plan using the Multiplan System and an X-sight spine tracking system was used to track lesions. The determination of radiotherapy dosage was based on factors such as histopathology, tumor location, and the tolerance dose of adjacent organs.

Treatment outcome assessment was assessed according to the Revised Response Evaluation Criteria in Solid Tumors (RECIST; version 1.1) [17], which divided the lesions into four groups: complete response (CR), partial response (PR), progressive disease (PD), and stable disease (SD). To facilitate the analysis, we classified the lesions into the PD group and non-PD group (including CR, PR, and SD).

### Clinical data and conventional imaging features collection

We collected clinical information comprising age, sex, location of the primary tumor (lung carcinoma /abdominal tumor/others), the number of spinal lesions (single/multiple), the presence of lymph node metastasis, the presence of visceral metastasis, the dose of radiation (30–40 Gy/5 fractions, 24–30 Gy/3 fractions, 18–24 Gy/1 fraction, or others), pain score (11-point numerical rating scale; 0–3/4–6/7–10) [18], Karnofsky performance status (KPS) score (80–100/0–70) [19], and spine instability neoplastic score (SINS; 0–6/7–12/13–18) [20].

The following conventional imaging features were collected: location (sacral/lumbar/thoracic/cervical), pattern of bone destruction (osteoblastic/osteolytic/ mixed), presence of a soft-tissue mass, involvement of the vertebral body, involvement of the pedicle, involvement of the lamina, presence of vertebral compression, and epidural spinal cord compression scale (Bilsky scale; 0–1/2–3) [21].

### Tumor segmentation

Two radiologists with over 5 years of experience, who were blinded to treatment outcomes, manually performed the segmentation of the tumor. The Digital Imaging and Communications in Medicine (DICOM) images were imported into the uAI Research Portal platform 1.1 (United Imaging Intelligence, Co., Ltd.) to perform image segmentation.

The region of interest (ROI) should include as much of the visible gross tumor as possible. Initially, the ROI was manually delineated on sagittal FS-T2WI. Subsequently, the ROI was replicated on sagittal T1WI and sagittal T2WI sequences and adjusted manually to ensure accuracy by redefining any problematic ROI.

### Image pre-processing and feature extraction

In the image pre-processing stage, we used B-spline interpolation resampling for image voxel size normalization, and all image sets were resampled to isotropic voxel size of 1 × 1 × 1 mm. *Z*-score intensity normalization was applied to reduce the impact of variability in image intensities on the stability of the radiomics features.

Feature extraction was performed using the uAI Research Portal platform 1.1 (United Imaging Intelligence, Co., Ltd.). Most features defined in this platform comply with feature definitions as described by the Image Biomarker Standardization Initiative (IBSI) [22].

The following classes of radiomics features were obtained from the original images: (1) First Order Statistics (19 features); (2) Shape-based (3D) (16 features); (3) Shape-based (2D) (10 features); (4) Gray Level Co-occurrence Matrix (GLCM, 24 features); (5) Gray Level Run Length Matrix (GLRLM, 16 features); (6) Gray Level Size Zone Matrix (GLSZM, 16 features); (7) Neighbouring Gray Tone Difference Matrix (NGTDM, 5 features); (8) Gray Level Dependence Matrix (GLDM, 14 features).

Furthermore, in order to increase the dimensionality of the dataset and improve the predictive performance of the models, we applied 17 different filters, including AdditiveGaussiannoise, Bilateral, BinomialBlurImage, BoxMean, BoxSigmaImage, CurvatureFlow, DiscreteGaussian, LaplacianSharpening, Mean, Median, Normalize, Recursive Gaussian, ShotNoise, SmoothingRecursiveGaussian, SpeckleNoise, LoG, and Wavelet to generate filtered images from the original ones. All classes of features, except for shape-based features, were computed on both the original and filtered images.

### Feature selection and prediction model development

Feature selection includes several steps. First, to ensure inter-observer reliability for the features extracted from the ROIs drawn by two radiologists, intraclass correlation coefficients (ICCs) were analyzed. Features with an ICC > 0.75 were deemed reliable and could be selected for model construction. Next, the minimal-redundancy-maximal-relevance (mRMR) framework was used to identify the most relevant features to tumor classification and eliminate redundant features [23]. This produced the top 100 highly relevant and least redundant features. Finally, we used the least absolute shrinkage and selection operator (LASSO) regression model, incorporating 10-fold cross-validation, to select features with nonzero coefficients [24]. In addition to performing feature selection on each sequence (T1WI, T2WI, and FS-T2WI sequences), feature selection was also performed across all three combined sequences (ALL sequences).

The final selected radiomics features were applied to 13 ML algorithms including AdaBoost, XGBoost, bagging decision trees (Bagging), decision tree classifiers (DT), gaussian processes (GP), gradient boosted decision trees (GBDT), k-nearest neighbor (KNN), logistic regression (LR), partial least squares discriminant analysis (PLS-DA), quadratic discriminant analysis (QDA), random forest (RF), stochastic gradient descent (SGD), and support vector machine (SVM) to construct the radiomics prediction models.

**Fig. 1:**
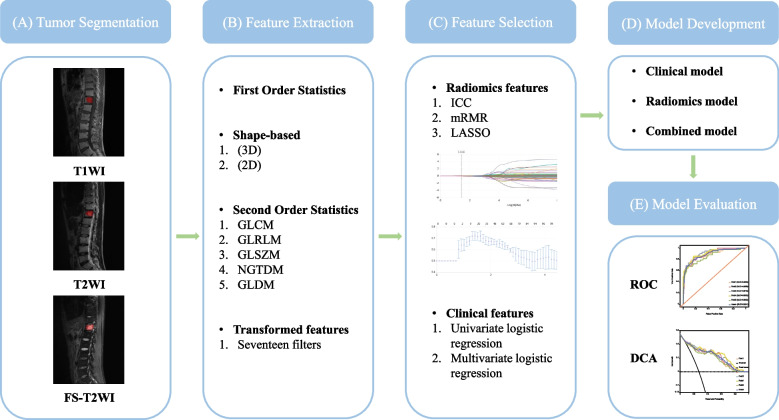
The workflow of prediction model construction. **a** Tumor segmentation was performed on T1WI, T2WI, and FS-T2WI. **b** Quantitative features were extracted from each ROI. **c** Feature selection was conducted to reduce feature dimensionality and enhance prediction performance. **d** and **e** Three types of prediction models were constructed and evaluated

Next, we incorporated clinical, conventional imaging, and radiomics features into the ML algorithms to construct the combined model. The workflow of the predictive model construction is shown in Fig. 1.

### Statistical analysis

The mean ± standard deviation (SD) was used to describe variables with a normal distribution. Median values and ranges were used to describe variables with a non-normal distribution. Proportions described categorical variables. Upon analysis using univariate logistic regression, we selected clinical variables with *p* < 0.20, which were carried forward for analysis using multivariate logistic regression. Odds ratios (ORs) with 95% confidence intervals (CIs) were obtained for each clinical variable. The performances of each model were compared using the area under the curve (AUC) of the receiver operating characteristic (ROC) curve. Additionally, we carried out decision curve analysis (DCA) at various threshold probabilities to assess the net benefits of each model and to determine their clinical applicability. SPSS software version 26.0 (IBM Corp.) and R version 4.2.0 (R Foundation for Statistical Computing) were used to perform the statistical analyses. Statistical significance was considered at *p*-values < 0.05.

## Results

### Study cohort

**Fig. 2:**
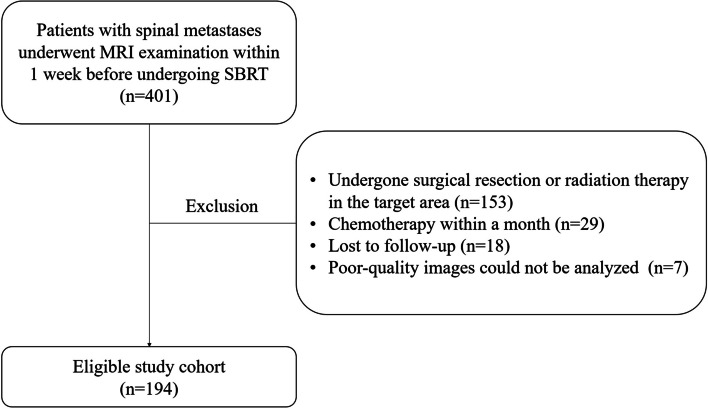
Inclusion and exclusion flowchart

We included 194 patients in the study, based on the inclusion and exclusion criteria (Fig. 2). The study population comprised 108 males and 86 females (mean age = 56.4 ± 15.3 years). There were 142 (73.2%) lesions in the non-PD group and 52 (26.8%) lesions in the PD group. Table 1 provides the detailed clinical and conventional imaging features for the entire cohort.

**Table 1:** Summary of clinical and conventional imaging features

		Non-PD group 142 (73.2%)	PD group 52(26.8%)
**Clinical features**			
Age (years)	Median (range)	59 (6–85)	58 (15–88)
Gender (*n*, %)	Male	76 (53.5%)	32 (61.5%)
	Female	66 (46.5%)	20 (38.5%)
Primary histology (*n*, %)	Lung	49 (34.5%)	16 (30.8%)
	Abdominal	48 (33.8%)	24 (46.2%)
	Other	45 (31.7%)	12 (23.1%)
No. of spinal lesions (*n*, %)	Single	106 (74.6%)	29 (55.8%)
	Multiple	36 (25.4%)	23 (44.2%)
Lymphatic metastasis (*n*, %)	No	115 (81.0%)	34 (65.4%)
	Yes	27 (19.0%)	18 (34.6%)
Visceral metastasis (*n*, %)	No	134 (94.4%)	46 (88.5%)
	Yes	8 (5.6%)	6 (11.5%)
Dose/fraction (*n*, %)	18–24 Gy/1f	21 (14.8%)	7 (13.5%)
	24–30 Gy/3f	69 (48.6%)	22 (42.3%)
	30–40 Gy/5f	42 (29.6%)	17 (32.7%)
	Other	10 (7.0%)	6 (11.5%)
Pain score	0–3	29 (20.4%)	4 (7.7%)
	4–6	71 (50.0%)	24 (46.2%)
	7–10	42 (29.6%)	24 (46.2%)
KPS score (*n*, %)	0–70	64 (45.1%)	35 (67.3%)
	80–100	78 (54.9%)	17 (32.7%)
SINS	0–6	48 (33.8%)	11 (21.2%)
	7–12	87 (61.3%)	35 (67.3%)
	13–18	7 (4.9%)	6 (11.5%)
**Conventional imaging features**			
Location (*n*, %)	Cervical	26 (18.3%)	13 (25.0%)
	Thoracic	69 (48.6%)	23 (44.2%)
	Lumbar	43 (30.3%)	13 (25.0%)
	Sacral	4 (2.8%)	3 (5.8%)
Bone destruction pattern (*n*, %)	Osteolytic	109 (76.8%)	37 (71.2%)
	Osteoblastic	14 (9.9%)	5 (9.6%)
	Mixed	19 (13.4%)	10 (19.2%)
Soft-tissue mass (*n*, %)	No	31 (21.8%)	9 (17.3%)
	Yes	111 (78.2%)	43 (82.7%)
Vertebral body involvement (*n*, %)	No	10 (7.0%)	4 (7.7%)
	Yes	132 (93.0%)	48 (92.3%)
Pedicle involvement (*n*, %)	No	62 (43.7%)	18 (34.6%)
	Yes	80 (56.3%)	34 (65.4%)
Lamina involvement (*n*, %)	No	89 (62.7%)	30 (57.7%)
	Yes	53 (37.3%)	22 (42.3%)
Compression fracture (*n*, %)	No	115 (81.0%)	35 (67.3%)
	Yes	27 (19.0%)	17 (32.7%)
Bilsky scale (*n*, %)	0–1	109 (76.8%)	29 (55.8%)
	2–3	33 (23.2%)	23 (44.2%)

*KPS* Karnofsky performance status, *SINS* Spine instability neoplastic score

### Clinical and conventional imaging feature-based model performance

**Fig. 3:**
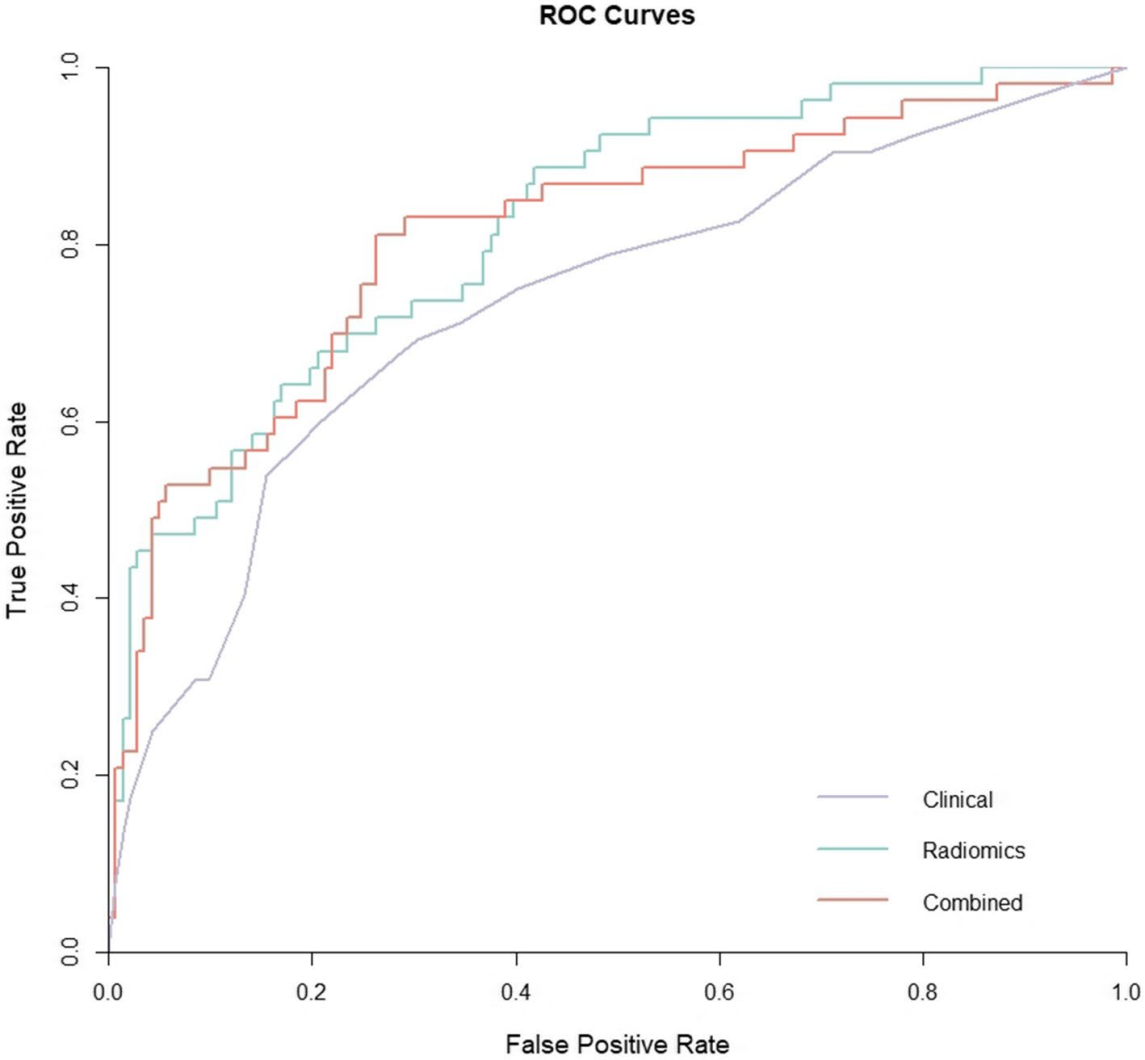
The ROC curves of the three models in test set. The radiomics and combined models outperform the clinical model significantly, with the combined model showing a slight improvement over the radiomics model

Univariate and multivariate logistic regression analyses indicated that the number of spinal lesions, pain score, KPS score, and Bilsky grade were independent predictors of PD (see Table 2 for details). PD was more likely in patients with multiple spinal lesions (OR = 2.13, 95% CI: 1.01–4.52, *p* = 0.048), a high pain score (OR = 4.839, 95% CI: 1.40–16.79, *p* = 0.013), a low KPS score (OR = 2.17, 95% CI: 1.04–4.52, *p* = 0.038), and a high Bilsky grade (OR = 2.49, 95% CI: 1.17–5.28, *p* = 0.018). The clinical prediction model was constructed using these four features. The AUC for the clinical prediction model in predicting PD was 0.733 (Fig. 3).

**Table 2:** Univariate and multivariate logistic regression analyses for selecting clinical and conventional imaging features

	Univariate analysis		Multivariate analysis	
	OR (95% CI)	*p*-value	OR (95% CI)	*p*-value
**Clinical features**				
Age (years)	1.008 (0.986–1.030)	0.483		
Gender (*n*, %)	0.720 (0.376–1.377)	0.320		
Primary histology (*n*, %)	0.925 (0.619–1.383)	0.704		
No. of spinal lesions (*n*, %)	2.335 (1.201–4.542)	0.012*	2.135 (1.010–4.514)	0.047*
Lymphatic metastasis (*n*, %)	2.255 (1.110–4.580)	0.025*	1.870 (0.851–4.109)	0.119
Visceral metastasis (*n*, %)	2.185 (0.720–6.631)	0.168		
Dose/fraction (*n*, %)	1.220 (0.829–1.796)	0.314		
Pain score	1.904 (1.164–3.113)	0.010*	1.970 (1.163–3.339)	0.012*
KPS score (*n*, %)	2.509 (1.288–4.889)	0.007*	2.095 (1.019–4.307)	0.044*
SINS	1.871 (1.039–3.369)	0.037*	1.401 (0.695–2.824)	0.346
**Conventional imaging features**				
Location (*n*, %)	1.278 (0.850–1.923)	0.239		
Bone destruction pattern (*n*, %)	1.226 (0.809–1.856)	0.337		
Soft-tissue mass (*n*, %)	1.334 (0.587–3.034)	0.491		
Vertebral body involvement (*n*, %)	0.909 (0.272–3.035)	0.877		
Pedicle involvement (*n*, %)	1.464 (0.756–2.834)	0.258		
Lamina involvement (*n*, %)	1.231 (0.645–2.351)	0.528		
Compression fracture (*n*, %)	2.069 (1.012–4.230)	0.046*	1.447 (0.653–3.207)	0.363
Bilsky scale (*n*, %)	2.620 (1.338–5.128)	0.005*	2.481 (1.178–5.225)	0.017*

*KPS* Karnofsky performance status, *SINS* Spine instability neoplastic score; *p < 0.05

### Radiomics model and combined model performances

A total of 2264 features were generated for each ROI. According to the standard of ICC > 0.75, rates of stable features for T1WI, T2WI, and FS-T2WI sequences were 66.3%, 81.6%, and 79.0%, respectively.

Overall, the radiomics model established using features from all three MRI sequences outperformed those based on a single sequence. The optimal models constructed based on T1WI, T2WI, and FS-T2WI sequences achieved AUC values of 0.779 (QDA), 0.823 (GP), and 0.745 (QDA) respectively. The optimal model based on ALL sequences attained an AUC of 0.825 (GP, Fig. 3).

**Fig. 4:**
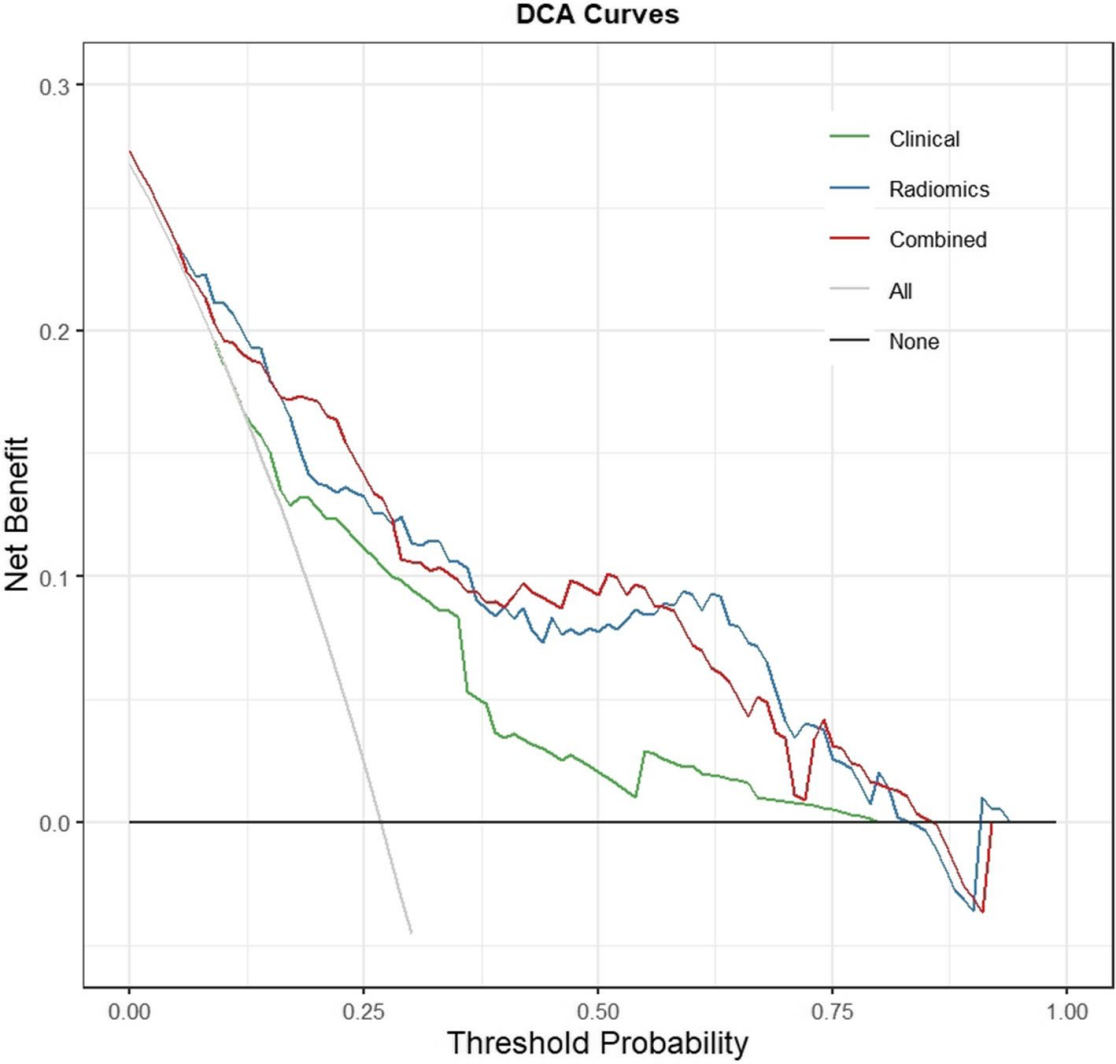
Decision curves for three models in the test set. Decision curve analysis demonstrates that the curves of the clinical, radiomics, and combined models all appear above the reference lines, indicating that these models provide a net benefit to improve clinical decision-making for patients. The radiomics and combined models exhibit higher net benefit compared with that of the clinical model

After incorporating clinical features, the performance of the combined model improved slightly. The best-performing model was based on ALL sequences using the GP algorithm (AUC = 0.828, Fig. 3). The diagnostic performance of the optimal ML classifier for each model is shown in Table 3. Decision curves revealed that the combined model exhibited the greatest net benefit in predicting PD (Fig. 4).

**Table 3:** Discrimination performance of all the models

Model	Optimal ML classifier	AUC	Sensitivity	Specificity	Accuracy	Precision
Clinical						
T1WI	QDA	0.779	0.436	0.936	0.799	0.738
T1WI + Clinical	SVM	0.816	0.478	0.895	0.778	0.652
T2WI	GP	0.823	0.460	0.944	0.814	0.751
T2WI + Clinical	GP	0.808	0.457	0.965	0.814	0.798
FS-T2WI	QDA	0.745	0.195	0.972	0.763	0.733
FS-T2WI + Clinical	LR	0.828	0.598	0.880	0.804	0.642
ALL	GP	0.825	0.473	0.957	0.825	0.830
ALL+ Clinical*	GP	0.828	0.511	0.950	0.830	0.813

*ML* Machine learning, *AUC* Area under the curve, *ALL* T1WI + T2WI + FS-T2WI sequences, *QDA* Quadratic discriminant analysis, *SVM* Support vector machine; *GP* Gaussian processes, *LR* Logistic regression; *best model

## Discussion

Predicting treatment outcomes for spinal metastases undergoing SBRT is challenging; however, certain clinical and radiological features show potential predictive value. Our research identified the Bilsky grade, KPS score, pain score, and the number of spinal lesions as independent PD predictors.

The number of spinal lesions affected treatment outcome: patients with multiple spinal lesions had a higher risk of PD compared to those with a single lesion, which agreed with previously published results [10, 11]. As a local treatment, SBRT is particularly suitable for patients with a single spinal metastasis. Tree et al. [25] reviewed existing evidence and recommended that SBRT should be considered for patients with isolated metastases. We observed that compared with that of patients with lower pain scores, those with higher scores were more prone to PD, consistent with prior research [10]. We speculated that the reason for this phenomenon might be related to the stage of the tumor, because pain is typically not significant in the early stages of the tumor, while it becomes intense in the advanced stages. Therefore, patients with more severe pain are more likely to experience PD. In addition, pain-induced physical limitations, emotional instability, sleep disorders, and dietary issues might also affect patient prognosis. We observed a higher probability of PD in patients with lower KPS scores, consistent with previous research [12, 26]. The KPS score reflects the patient’s condition, with a lower score often indicating poorer overall health. This can be a sign of serious underlying health issues or symptoms. Consequently, patients with lower KPS scores might experience more difficulties and complications during SBRT, leading to a less favorable response to treatment and a worse prognosis. Based on T2WI, the Bilsky grade assesses the severity of spinal stenosis. Patients with Bilsky grades 2–3, indicating severe spinal stenosis, tended to experience PD more frequently than those with Bilsky grades 0–1 (mild stenosis), consistent with a previous study [11]. This might have been caused by the presence of larger tumors and their proximity to the spinal cord, which would limit the radiation dose. Consequently, SBRT might not be suitable for patients with severe spinal stenosis, particularly those with Bilsky grade 3 and significant neurological symptoms. Surgical treatment should be considered in such cases [27].

The clinical prediction model built based on the selected features showed a decent predictive value (AUC = 0.733). In comparison, the radiomics models constructed using MRI demonstrate better performance (AUC = 0.745–0.825). Radiomics techniques can extract a vast amount of information, providing a more comprehensive and detailed description of lesion characteristics [28]. By capturing subtle changes within the lesion, radiomics features can provide a more precise depiction of the complex physiopathological mechanisms of tumors. Therefore, the radiomics models based on MRI have significant value in predicting the treatment outcome of SBRT. Furthermore, when clinical features were incorporated, the combined models contained more valid information to predict PD, leading to a further improvement in performance (AUC = 0.828).

ML algorithms play a vital role as indispensable tools in radiomics. By extensively learning and training on a vast dataset, ML algorithms can reveal the association between image biomarkers and treatment outcomes [29]. When choosing the most suitable ML algorithm, it is vital to account for factors including data characteristics, task type, data scale, algorithm efficiency, and predictive performance [30]. Therefore, the optimal choice depends on specific circumstances. In our study, we utilized 13 ML algorithms to construct the predictive models. We found that the performances of the GP and QDA algorithms were better than those of other ML algorithms. We speculated that both GP and QDA share common characteristics of being able to handle nonlinear relationships between features and are applicable to small sample sizes without requiring large amounts of training data [31, 32].

As far as we know, this was the first study to employ MRI-based radiomics models to predict the treatment outcome post-SBRT in patients with spinal metastases. Previous studies have mainly investigated the association between treatment outcomes and clinical features [11, 12, 33]. Additionally, one study employed CT-based radiomics to predict patient’s pain response [34].

There were limitations associated with the present study. Firstly, this was a single-center study involving a limited number of cases. Sample size can affect the performance and generalization ability of ML algorithms. Therefore, large-scale multicenter studies should be carried out to gain more corroborative evidence for clinical applications. Secondly, we could only construct models based on MRI features because the majority of the patients did not receive CT scans before treatment. This was because MRI provides better visualization of spinal metastases due to its high soft-tissue resolution and optimal target volume delineation for SBRT compared with CT. From an economic aspect, except for those initially identified with metastases on CT, only pretreatment MRI was performed in our cohort. The value of radiomics models based on CT, as well as other imaging examinations, such as PET and functional MRI, deserves further exploration.

## Conclusion

In conclusion, MRI is commonly utilized as an imaging modality before SBRT, enabling a comprehensive evaluation of target lesions. Through the analysis of radiomic features in MRI, our constructed models could predict treatment outcomes following SBRT in spinal metastases. Furthermore, the incorporation of clinical features further improved the performance of the models. These predictive models should aid clinicians’ decision-making and will contribute to improved prognosis of patients suffering from spinal metastases.

## Data Availability

The datasets used and/or analyzed during the current study are available from the corresponding author on reasonable request.

## Abbreviations

Bagging: Bagging decision trees
CI: Confidence interval
DT: Decision tree classifiers
FS-T2WI: Fat-suppression T2-weighted image
GBDT: Gradient-boosted decision trees
GP: Gaussian processes
ICC: Intraclass correlation coefficient
KNN: k-nearest neighbor
KPS: Karnofsky performance status
LR: Logistic regression
ML: Machine learning
OR: Odds ratio
PD: Progressive disease
PLS-DA: Partial least squares discriminant analysis
QDA: Quadratic discriminant analysis
RF: Random forest
SBRT: Stereotactic body radiotherapy
SGD: Stochastic gradient descent
SVM: Support vector machine
T1WI: T1-weighted image
T2WI: T2-weighted image
